# Supplementary material for: MetaRibo-Seq measures translation in microbiomes
Source: Nat Commun. 2020 Jun 29;11:3268. doi: 10.1038/s41467-020-17081-z (PMC7324362; doi:10.1038/s41467-020-17081-z)
Supplement: Supplementary file 10 — Supplementary Data 7 [file 41467_2020_17081_MOESM10_ESM.zip › File2/Confidence_VeryHigh_Taxonomy/164547_out.krona.html]

Javascript must be enabled to view this page.

members
magnitude
magnitudeUnassigned
count
unassigned
taxon
rank

164547\_out

5

2
5
superkingdom

1239
phylum
5

186801
5
class

5
order
186802

1
family
186803

genus
1
1506553

species

SRS013687\_contig\_number\_contig-100\_24863.69806
1
1946695

4
family
31979

1485
4
genus

1262777
species
4

SRS024435\_contig\_number\_contig-100\_2083.84067SRS047014\_contig\_number\_contig-100\_2295.128806SRS075773\_contig\_number\_11635SRS075821\_contig\_number\_contig-100\_7534.7535
